# Nomograms for Predicting Medical Students' Perceptions of the Learning Environment: Multicenter Evidence From Medical Schools in China

**DOI:** 10.3389/fpubh.2022.825279

**Published:** 2022-04-29

**Authors:** Zhitong Zhou, Runzhi Huang, Guoyang Zhang, Meiqiong Gong, Shuyuan Xian, Huabin Yin, Tong Meng, Xiaonan Wang, Yue Wang, Wenfang Chen, Chongyou Zhang, Erbin Du, Min Lin, Xin Liu, Qing Lin, Shizhao Ji, Hongbin Wu, Zongqiang Huang, Jie Zhang

**Affiliations:** ^1^Department of Gynecology, Shanghai First Maternity and Infant Hospital, Tongji University School of Medicine, Shanghai, China; ^2^Key Laboratory of Spine and Spinal Cord Injury Repair and Regeneration of Ministry of Education, Orthopaedic Department of Tongji Hospital, Tongji University School of Medicine, Shanghai, China; ^3^Tongji University School of Medicine, Shanghai, China; ^4^School of Education, Shanghai Jiao Tong University, Shanghai, China; ^5^Office of Educational Administration, Shanghai University, Shanghai, China; ^6^Department of Orthopedics, Shanghai General Hospital, School of Medicine, Shanghai Jiao Tong University, Shanghai, China; ^7^Department of Epidemiology and Health Statistics, School of Public Health, Capital Medical University, Beijing, China; ^8^Department of Health Statistics, School of Public Health, The Forth Military Medical University of PLA, Xi'an, China; ^9^Faculty of Medicine, Jinggangshan University, Ji'An, China; ^10^Center of Science and Technology Research and Development and Industrial Management, Harbin Medical University, Heilongjiang, China; ^11^Frist Clinical Medical College, Mudanjiang Medical University, Mudanjiang, China; ^12^Mental Health Education and Consultation Center, Chongqing Medical University, Chongqing, China; ^13^Department of Rheumatology and Immunology, Second Affiliated Hospital of Naval Medical University, Shanghai, China; ^14^Department of Human Anatomy, Laboratory of Clinical Applied Anatomy, School of Basic Medical Sciences, Fujian Medical University, Fuzhou, China; ^15^Department of Burns, The First Affiliated Hospital of Naval Medical University, Shanghai, China; ^16^Institute of Medical Education/National Centre for Health Professions Education Development, Peking University, Beijing, China; ^17^Department of Orthopedics, The First Affiliated Hospital of Zhengzhou University, Zhengzhou, China

**Keywords:** medical students, perceptions, learning environment, nomograms, prediction

## Abstract

Medical students' perceptions of the medical school learning environment (MSLE) have an important impact on their professional development, and physical and mental health. Few studies reported potential factors that influenced medical students' perceptions of MSLE. Thus, the main goal of this study was to identify influencing factors for medical students' perception levels of MSLE. The perception levels of MSLE were assessed by the Johns Hopkins Learning Environment Scale. The univariate and multivariate logistic regression analyses were performed to identify significant predictors for the perceptions of MSLE. The nomograms were established to predict medical students' perception levels of MSLE. In the multivariate logistic regression model, gender, university category, grade, mother education level, learning environment of schools, interests in medicine, and Kolb learning experience were significantly associated with medical students' perceptions of MSLE. Correspondently, the nomograms were built based on significant variables identified by the univariate logistic regression analysis. The validation of the nomograms showed that the model had promising predictive accuracy, discrimination, and accordance (area under the curve (AUC) = 0.751). This study identified influencing factors of medical students' perceptions of MSLE. It is essential to implement corresponding interventions to improve medical students' perceptions.

## Introduction

Learning environment (LE) can be conceptualized as physical, social, and psychological contexts in which students learn (1). Students can perceive LE, and the perceptions will influence their behavior in the school (1). In the Standards for Accreditation of Medical Education Programs, the Liaison Committee on Medical Education (LCME) underscored the importance of the LE for the development of explicit and appropriate professional behaviors in medical students (2). Studies reported that a supportive LE can enhance medical students' academic performance and wellbeing (3, 4). Furthermore, students supported in the psychological environment would be more likely to respond to academic stressors and challenges in positive ways (5). However, an unsupportive LE may cause burnout (6), emotional exhaustion, depersonalization, and lower quality of life for medical students (7). Thus, LE is an important determinant for the behavior of medical students. Medical students experience greater academic and psychological stress than their peers and the general people (8, 9), so a favorable medical school learning environment (MSLE) extremely matters for medical students' learning and wellbeing.

One method for evaluating MSLE is to assess students' perceptions of MSLE. The perceptions may be affected by many factors (10). Thus, studies investigating factors that affect medical students' perceptions of MSLE are warranted to engage medical students' learning, promote their academic development, and improve the quality of medical education. However, research to date has not yet explored predictive factors of MSLE based on a large sample of multiple centers in China.

The Johns Hopkins Learning Environment Scale (JHLES) is an approach to assessing students' perceptions of MSLE (11). Items of the JHLES describe social, relational, and academic processes of medical school that support students' professional formation (11). The nomogram is an important tool to estimate the prognosis of oncology and other diseases (12). Its primary advantage is that it can predict individualized risk based on patient and disease characteristics (12). The nomogram transforms the complex regression equation into a visual graph, making the results of the predictive model more readable.

Thus, the main purpose of this article is to determine significant predictors of medical students' perceptions of MSLE. Then, the nomograms were built to predict medical students' perception levels of MSLE.

## Materials and Methods

### Sample Source and Data Extraction

This study was approved by the Ethics Committee of Tongji Hospital, Tongji University School of Medicine.

We launched a cross-sectional study from 11 universities in mainland China from 20 February 2020 to 31 March 2020. We randomly selected medical students from these universities, including 985 Project Universities (Peking University, Tongji University), 211 Project Universities (Zhengzhou University), military universities (Air Force Medical University), the First Batches of Medical Universities (Capital Medical University, Harbin Medical University, Fujian Medical University, Chongqing Medical University, and Southwest Medical University), the Second Batches of Medical Universities (Mudanjiang Medical College) and Non-985/211 Project Universities (Jinggangshan University).

Firstly, to evaluate the quality and readability of the questionnaire, 20 students in grades 4 and 5 of Tongji university school of medicine were randomly selected to conduct a pilot study, and the questionnaire was revised according to each student's feedback. Then, we integrated questionnaire information into Wenjuanxing (https://www.wjx.cn/), an online tool for the questionnaire survey, and the link of the questionnaire was sent to corresponding heads of 11 medical schools of universities determined cooperative relationship. Finally, we performed a stratified cluster random sampling based on grade. In each grade, 1 to 2 classes were randomly selected by lottery, and all students in each class were selected to fill out the questionnaire. The questionnaires were issued in a unified format. After the questionnaires were collected, the questionnaires with outliers and missing values were eliminated and recorded as invalid questionnaires.

### Instrument

In this study, Johns Hopkins Learning Environment Scale (JHLES) was utilized to assess medical students' perceptions of MSLE (Supplementary Table S1). The scale consisted of seven subscales (Community of peers, Faculty relationships, Academic climate, Meaningful engagement, Mentoring, Inclusion and safety, and Physical space) and a total of 28 items, and we used a five-point Likert response scale that ranged from strongly disagree (1) to strongly agree (5) to assess each item (11). The higher score showed a more positive perception of MSLE. A previous study demonstrated that the scale had good reliability and validity (13). In this study, the scale also showed favorable internal reliability (total Cronbach's α: 0.949; seven domains Cronbach's α: 0.863–0.924) and structure validity (KMO value = 0.965, *p* < 0.001).

### Statistical Analysis

For descriptive statistical analysis, continuous variables were presented as mean (standard deviation, SD) or median (interquartile range) and categorical variables as number (percentage). JHLES scores were divided into low and high groups following the median value. The univariate logistic regression was performed to screen significant variables associated with JHLES scores. Then, these significant variables were used to construct the multivariate logistic regression model. Finally, the nomograms were built to predict the probability of a high JHLES score. The discrimination and calibration performances of nomograms were evaluated by receiver operating characteristic (ROC) and calibration curves. Decision curve analysis (DCA) was used to assess the net benefit of medical students.

All statistical analyses were performed using R version 3.6.1 (Institute for Statistics and Mathematics, Vienna, Austria) and SPSS20.0 (SPSS Inc., Chicago, IL, USA). A two-sided *p* < 0.05 was considered statistical significance.

## Results

### Sample Characteristics

**Figure 1:**
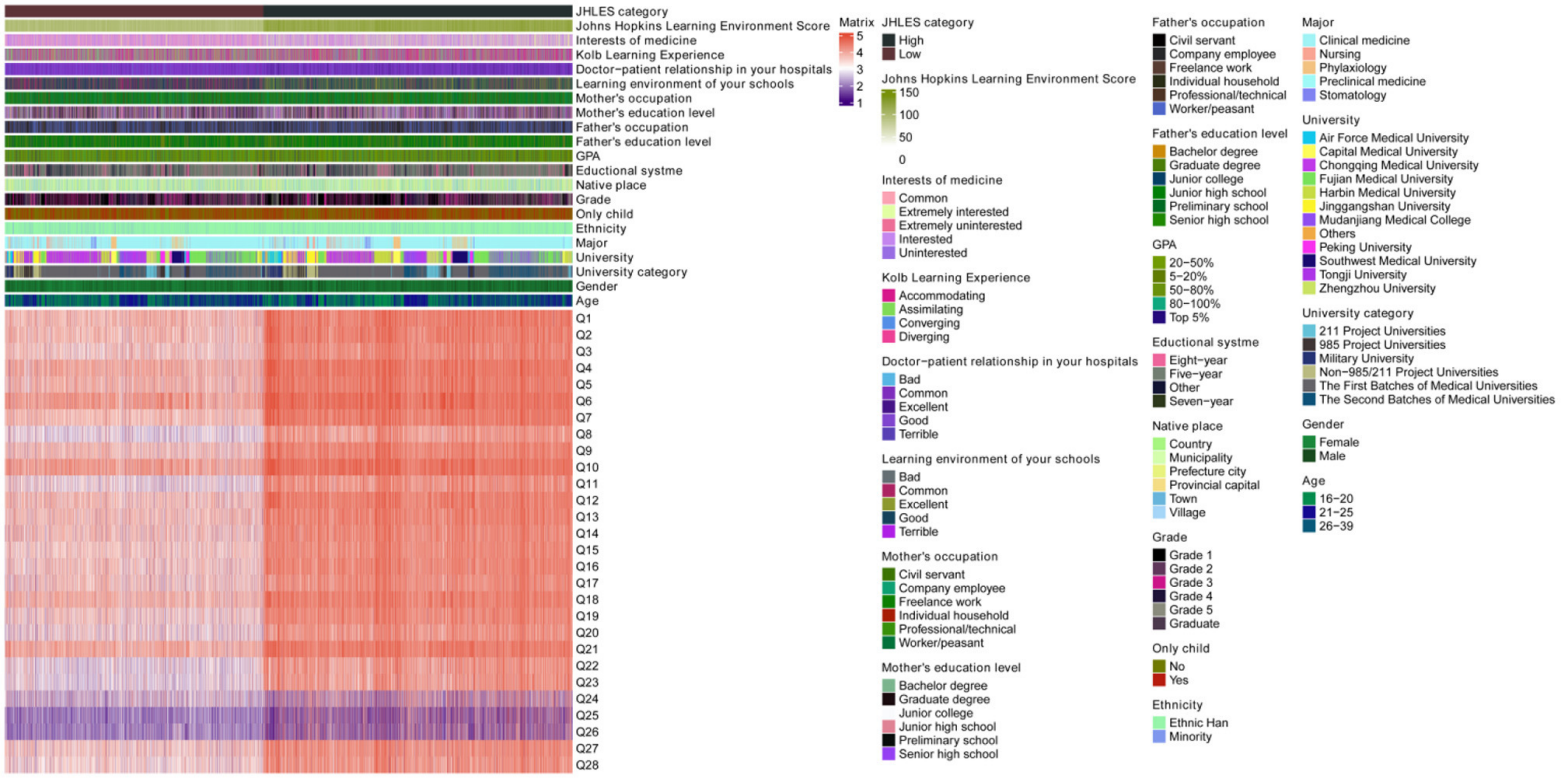
Heatmap of the baseline characteristics of the participants. GPA, grade point average; JHLES, Johns Hopkins Learning Environment Scale.

We received 10,901 questionnaires, but a total of 10,576 questionnaires could be used to further analysis. The age of the students was mainly concentrated in the 16–25 years (98.79%). Males were 20.48% more than females. Most students majored in clinical medicine (79.15%). There were more students in grade 1(35.19%), followed by grade 2 (18.78%). More than half of students had a 5-year educational system (69.74%). Most students' parents had a low education level. Most students had a good feeling about school LE (55.77% was good, 21.67% was excellent). Accommodation (33.77%) and assimilation (29.49%) were the preferred learning styles of medical students. Half of the students were interested in medicine (56.45%). About 54.46% of students had good perceptions of MSLE (Figure 1, Table 1).

**Table 1:** Characteristics of 10,576 participants.

**Variables**	**Number (percentage)**
**Age**	
16–20	5,715 (54.04)
21–25	4,733 (44.75)
26–39	128 (1.21)
**Gender**	
Male	4,205 (39.76)
Female	6,371 (60.24)
**University category**	
211 Project Universities	692 (6.54)
985 Project Universities	853 (8.07)
Military University	526 (4.97)
Non−985/211 Project Universities	720 (6.81)
The First Batches of Medical Universities	6,473 (61.20)
The Second Batches of Medical Universities	1,312 (12.41)
**University**	
Air Force Medical University	526 (4.97)
Capital Medical University	334 (3.16)
Chongqing Medical University	2,219 (20.98)
Fujian Medical University	2,533 (23.95)
Harbin Medical University	853 (8.07)
Jinggangshan University	706 (6.68)
Mudanjiang Medical College	1,304 (12.33)
Others	43 (0.41)
Peking University	369 (3.48)
Southwest Medical University	534 (5.05)
Tongji University	481 (4.55)
Zhengzhou University	674 (6.37)
**Major**	
Clinical medicine	8,371 (79.15)
Nursing	567 (5.36)
Phylaxiology	689 (6.52)
Preclinical medicine	652 (6.16)
Stomatology	297 (2.81)
**Ethnicity**	
Ethnic Han	9,893 (93.54)
Minority	683 (6.46)
**Only child**	
No	5,977 (56.51)
Yes	,4599 (43.49)
**Grade**	
Grade 1	3,722 (35.19)
Grade 2	1,986 (18.78)
Grade 3	1,639 (15.50)
Grade 4	1,843 (17.42)
Grade 5	1,254 (11.86)
Graduate	132 (1.25)
**Native place**	
Country	2,533 (23.95)
Municipality	1,484 (14.03)
Prefecture city	1,974 (18.67)
Provincial capital	1,088 (10.29)
Town	1,131 (10.69)
Village	2,366 (22.37)
**Educational system**	
Eight–year	1,281 (12.11)
Five–year	7,376 (69.74)
Other	1,639 (15.50)
Seven–year	280 (2.65)
**GPA**	
20–50%	3,744 (35.40)
5–20%	2,431 (22.99)
50–80%	2,640 (24.96)
80–100%	1,003 (9.48)
Top 5%	758 (7.17)
**Father's education level**	
Bachelor degree	1,235 (11.68)
Graduate degree	233 (2.20)
Junior college	1,104 (10.44)
Junior high school	3,721 (35.18)
Preliminary school	1,769 (16.73)
Senior high school	2,514 (23.77)
**Father's occupation**	
Civil servant	1,032 (9.76)
Company employee	1,057 (9.99)
Freelance work	2,062 (19.50)
Individual household	1,056 (9.98)
Professional/technical	1,103 (10.43)
Worker/peasant	4,266 (40.34)
**Mother's education level**	
Bachelor degree	910 (8.60)
Graduate degree	163 (1.54)
Junior college	977 (9.24)
Junior high school	3,241 (30.65)
Preliminary school	3,126 (29.56)
Senior high school	2,159 (20.41)
**Mother's occupation**	
Civil servant	599 (5.66)
Company employee	1,206 (11.40)
Freelance work	2,816 (26.63)
Individual household	770 (7.28)
Professional/technical	1,308 (12.37)
Worker/peasant	3,877 (36.66)
**Learning environment of your schools**	
Bad	116 (1.10)
Common	2,210 (20.89)
Excellent	2,292 (21.67)
Good	5,898 (55.77)
Terrible	60 (0.57)
**Doctor–patient relationship in your hospitals**	
Bad	117 (1.11)
Terrible	45 (0.42)
Common	2,753 (26.03)
Good	6,009 (56.82)
Excellent	1,652 (15.62)
**Kolb learning experience**	
Accommodating	3,572 (33.77)
Assimilating	3,119 (29.49)
Converging	1,734 (16.40)
Diverging	2,151 (20.34)
**Interests of medicine**	
Common	2,599 (24.57)
Extremely interested	1,781 (16.84)
Extremely uninterested	65 (0.62)
Interested	5,970 (56.45)
Uninterested	161 (1.52)
**JHLES category**	
High score (≥104)	5,760 (54.46)
Low score (<104)	4,816 (45.54)

*GPA, grade point average; JHLES, Johns Hopkins Learning Environment Scale*.

### The Univariate and Multivariate Logistic Regression Analysis

JHLES scores were divided into low and high groups following 104 score. First, we performed the univariate logistic regression analysis to select significant variables associated with JHLES score. Nine variables including gender, university category, only child, grade, native place, mother education level, current learning environment of schools, interest in medicine, Kolb learning experience showed statistical significance (*p* < 0.05) (Table 2). Then, nine variables were integrated into the multivariate logistic regression model. In the multivariate logistic regression model, males were associated with better perceptions of MSLE compared to females (odds ratio (OR) = 1.43, 95%CI = 1.31–1.57, *p* < 0.001). Non-985/211 Project Universities and the Second Batches of Medical Universities students had better perceptions (OR = 1.50, 95%CI = 1.19–1.90, *p* = 0.001; OR = 1.79, 95%CI = 1.44–2.22, *p* < 0.001, respectively), however, 985 project university students had the opposite result (OR = 0.74, 95%CI = 0.59–0.92, *p* = 0.008). Grade 5 was a protective factor for poor perceptions (OR = 1.25, 95%CI =1.08–1.45, *p* = 0.003), but grade 2 and grade 4 were risk factors for poor perceptions (OR = 0.85, 95%CI = 0.76–0.96, *p* = 0.01; OR = 0.81, 95%CI = 0.71–0.92, *p*= 0.001, respectively). Preliminary school education level of mothers was related to worse perceptions (OR = 0.79, 95%CI = 0.66–0.95, *p* = 0.013). Common, good and excellent LE of schools tended to have better perceptions (OR = 2.95, 95%CI = 1.76–5.26, *p* < 0.001; OR = 7.62, 95%CI = 4.56–13.51, *p* < 0.001; OR = 18.38, 95%CI = 10.88–32.85, *p* < 0.001, respectively). Interested and extremely interested of medicine were associated with better perceptions (OR = 2.02, 95%CI = 1.82–2.24, *p* < 0.001; OR = 4.45, 95%CI = 3.79–5.23, *p* < 0.001, respectively). Compared to accommodating, assimilating, converging and diverging learning experience had worse perceptions (OR = 0.70, 95%CI = 0.63–0.78, *p* < 0.001; OR = 0.78, 95%CI =0.69–0.89, *p* = 0.003; OR = 0.83, 95%CI = 0.74–0.94, *p* < 0.001, respectively) (Table 3).

**Table 2:** Univariate logistic regression analysis of JHLES scores.

**Variables**	**JHLES score**	
	**OR (95% CI)**	***P*-value**
Age	1.31 (0.82–2.09)	0.264
Gender	1.51 (1.39–1.63)	<0.001^*^
University category	0.70 (0.57–0.85)	<0.001^*^
Major	0.94 (0.79–1.11)	0.448
Ethnicity	0.90 (0.77–1.06)	0.204
Only child	1.18 (1.09–1.27)	<0.001^*^
Grade	0.74 (0.67–0.83)	<0.001^*^
Native place	0.82 (0.72–0.94)	0.003^*^
Educational system	1.08 (0.96–1.21)	0.217
GPA	1.04 (0.94–1.15)	0.438
Father education level	0.80 (0.61–1.06)	0.121
Father occupation	0.88 (0.74–1.05)	0.149
Mother education level	0.69 (0.50–0.97)	0.032^*^
Mother occupation	0.90 (0.74–1.10)	0.294
Learning environment of your schools	2.59 (1.54–4.37)	<0.001^*^
Doctor patient relationship in your hospitals	1.31 (0.87–1.98)	0.193
Interests of medicine	9.23 (7.97–10.68)	<0.001^*^
Kolb learning experience	0.69 (0.63–0.76)	<0.001^*^

*JHLES, Johns Hopkins Learning Environment Scale; GPA, grade point average; OR, odds ratio; CI, confidence interval*.

* *P < 0.05*.

**Table 3:** Multivariate logistic regression analysis of JHLES scores.

**Variables**	**JHLES scores**	
	**OR (95% CI)**	***P*-value**
**Gender**		
Female	1.00 (reference)	
Male	1.43 (91.31–1.57)	<0.001^*^
**University category**		
211 Project Universities	1.00 (reference)	
985 Project Universities	0.74 (0.59–0.92)	0.008 ^*^
Military University	1.13 (0.87–1.46)	0.363
Non-985/211 Project Universities	1.50 (1.19–1.90)	0.001^*^
The First Batches of Medical Universities	0.86 (0.72–1.03)	0.109
The Second Batches of Medical Universities	1.79 (1.44–2.22)	<0.001^*^
**Only child**		
No	1.00 (reference)	
Yes	1.04 (0.95–1.15)	0.400
**Grade**		
Grade 1	1.00 (reference)	
Grade 2	0.85 (0.76-0.96)	0.01^*^
Grade 3	0.90 (0.79–1.02)	0.102
Grade 4	0.81 (0.71–0.92)	0.001^*^
Grade 5	1.25 (1.08–1.45)	0.003^*^
Graduate	1.46 (0.97–2.21)	0.075
**Native place**		
Country	1.00 (reference)	
Municipality	0.96 (0.83–1.11)	0.537
Prefecture city	1.04 (0.91–1.19)	0.562
Provincial capital	0.99 (0.85–1.17)	0.945
Town	1.01 (0.86–1.18)	0.911
Village	1.12 (0.98–1.28)	0.088
**Mother education level**		
Bachelor degree	1.00 (reference)	
Graduate degree	0.73 (0.50–1.06)	0.102
Junior college	0.99 (0.81–1.22)	0.926
Junior high school	1.00 (0.84–1.20)	0.975
Senior high school	0.98 (0.82–1.17)	0.819
Preliminary school	0.79 (0.66–0.95)	0.013^*^
**Learning environment of your schools**		
Bad	1.00 (reference)	
Terrible	1.92 (0.84–4.40)	0.12
Common	2.95 (1.76–5.26)	<0.001^*^
Good	7.62 (4.56–13.51)	<0.001^*^
Excellent	18.38 (10.88–32.85)	<0.001^*^
**Interests of medicine**		
Common	1.00 (reference)	
Extremely uninterested	0.73 (0.38–1.34)	0.331
Uninterested	0.41 (0.26–0.62)	<0.001^*^
Interested	2.02 (1.82–2.24)	<0.001^*^
Extremely interested	4.45 (3.79–5.23)	<0.001^*^
**Kolb learning experience**		
Accommodating	1.00 (reference)	
Assimilating	0.70 (0.63–0.78)	<0.001^*^
Converging	0.78 (0.69–0.89)	0.003^*^
Diverging	0.83 (0.74–0.94)	<0.001^*^

*JHLES, Johns Hopkins Learning Environment Scale; OR, odds ratio; CI, confidence interval*.

* *P < 0.05*.

### Nomograms and Validation

**Figure 2:**
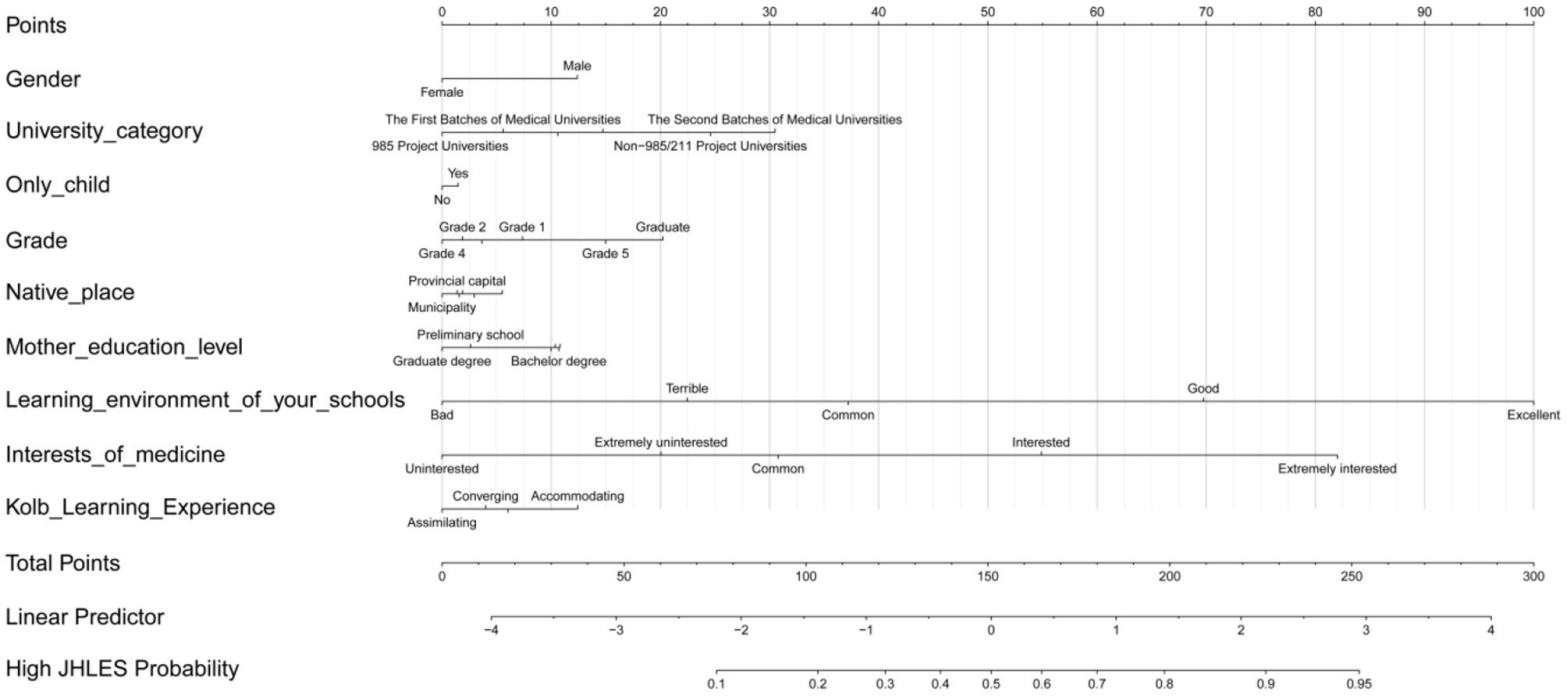
Nomograms of predicting medical students' perceptions of MSLE. MSLE, medical school learning environment; JHLES, Johns Hopkins Learning Environment Scale.

**Figure 3:**
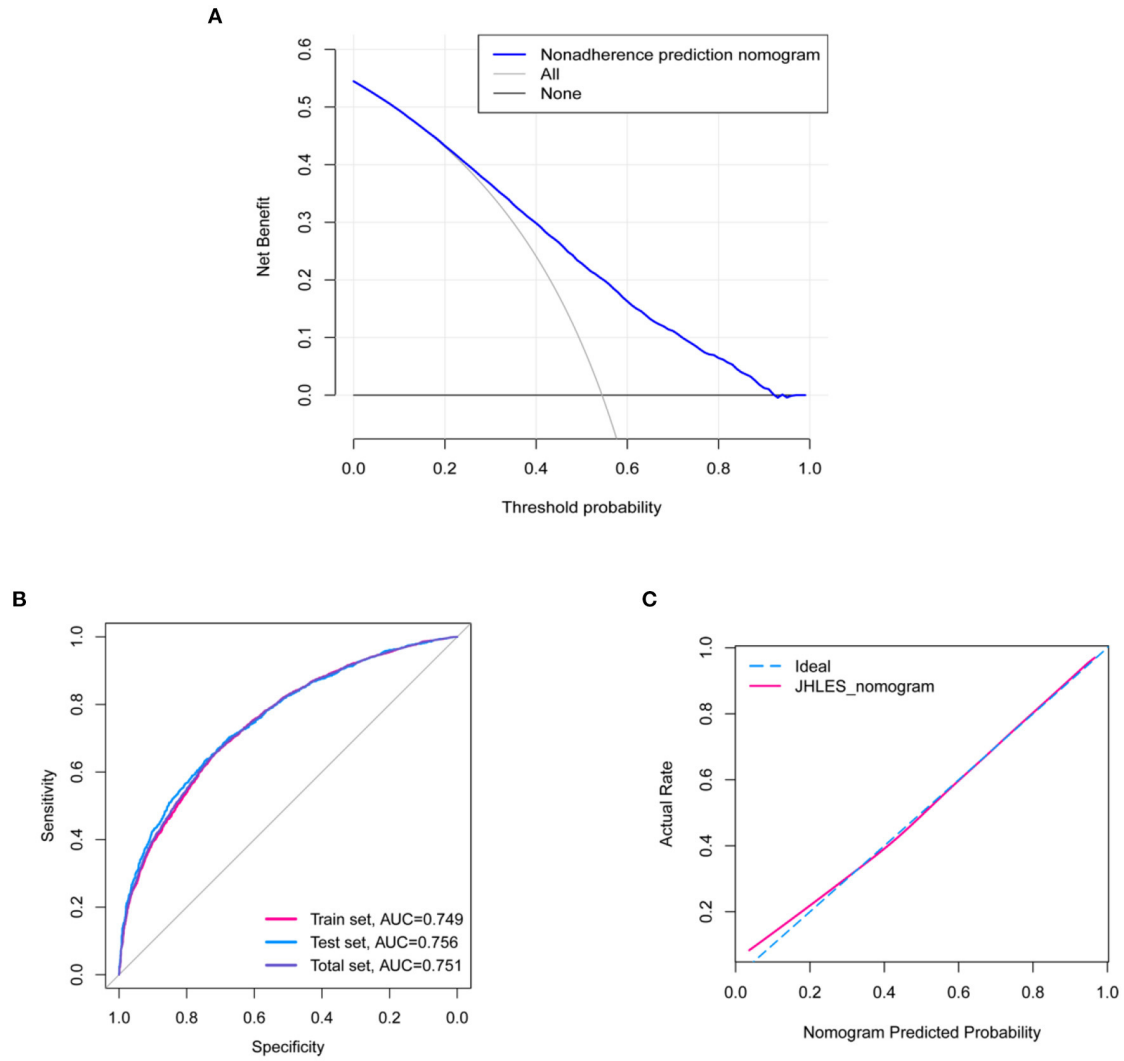
Validation of the nomograms. **(A)** DCA of the nomograms. Medical students had higher net benefit when high JHLES probability was >0.25. **(B)** The ROC curve of the nomograms. The ROC curve showed that the predictive model had potential predictive discrimination and accuracy (Total set AUC = 0.751, Train set AUC = 0.749, Test set AUC = 0.756). **(C)** The calibration curve of the nomograms. DCA, decision curve analysis; JHLES, Johns Hopkins Learning Environment Scale; ROC, receiver operating characteristic; AUC, area under the curve.

The nomograms were established based on statistically significant variables in the univariate logistic regression analysis (Figure 2). The internal validation of the nomograms was assessed by ROC and calibration curves. The DCA indicated that medical students had higher net benefits when the high JHLES probability was >0.25 (Figure 3A). Additionally, the results also showed the nomograms model had potentially predictive accuracy and discrimination (AUC = 0.751) (Figure 3B). Moreover, the AUCs in the train set and test set were similar, it further showed the model had good predictive power. Furthermore, we found that the nomograms were well-calibrated (Figure 3C), which suggested the predictive JHLES score had good accordance with the actual JHLES score.

## Discussion

MSLE plays an important role in medical students' learning outcomes. Favorable perceptions of MSLE may enhance academic achievement, empathy, wellbeing, and reduce burnout, and even distress of medical students (14). Thus, it is necessary to explore factors that affect medical students' perceptions of MSLE.

In this study, the JHLES was utilized to evaluate medical students' perceptions of MSLE. The univariate logistic regression analysis identified nine variables including gender, university category, only child, grade, native place, mother education level, learning environment of schools, interest in medicine, Kolb learning experience were associated with medical students' perceptions of MSLE (*p* < 0.05). Moreover, nine significant variables were integrated into the multivariable logistic regression model. The results indicated that the factors were beneficial for perceptions of MSLE, such as male, non-985/211 Project Universities, the Second Batches of Medical Universities, grade 5, common, good or excellent LE of school, and interested or extremely interested in medicine. Then, the nomograms were constructed based on nine variables to predict medical students' perception levels of MSLE. Eventually, validation of the nomograms showed the model had promising predictive power (AUC = 0.751) and accordance. What's more, medical students had higher net benefits when the high JHLES probability was >0.25.

Students' perceptions of MSLE were not only influenced by the school characteristics, but also by the student characteristics and family characteristics (10, 15). Our results showed that males had better perceptions of MSLE compared to females. Related studies also reported that the males had more positive perceptions of LE (16, 17). Nevertheless, results that there was no difference or the females had better perceptions than males have also been reported (18, 19). Thus, the relationship between gender and perceptions of MSLE still needs further investigation. We found that the university category was also a factor influencing students' perceptions. The students of 985 Project universities were more likely to have poor perceptions of MSLE. Relatively speaking, 985 universities in China mean high quality and high standards. Students have more competition and more pressure, which may lead to worse perceptions. In addition, compared to grade 1, students' perceptions of MSLE in grade 4 were worse. When students transited to the clinical internship environment, their perceptions of MSLE will deteriorate (10). Moreover, students might face dual stress of postgraduate exams and clinical internships in the 4th year. When they balance internships and exams, they do not receive better LE support from medical schools, which may affect their perceptions. However, students' perceptions in grade 5 were better. During the 2-year clinical internships stage, the perceptions were different, which indicated that the needs of students were different at different stages, so teachers should pay more attention to the needs of students during these 2 years to help them improve their perceptions (20). The mother's educational level in preliminary school was a risk factor for students' lower perception levels. Most students' mothers had lower educational levels in this study. Lower maternal education was associated with poor school performance of children (21). Parents with a higher education level may pay more attention to the comprehensive training of their children. Moreover, maternal behaviors were more likely to have an impact on children's behaviors and further affect their overall performance in the subsequent educational environment (22). The results showed individuals relatively subjectively believed that the better the LE of schools was, the better the perception was. In this study, we found that students who were more interested in medicine tended to have better perceptions of MSLE. Therefore, although some students who choose to study medicine may not be out of interest in medicine, it is also crucial for schools to provide a LE that is conducive to increasing students' interest in medicine. Kolb divides learning styles into four types: Accommodating, Assimilating, Converging, and Diverging (23). Learning style was a learner's preference for collecting, organizing and processing information and it was about how learners study (24, 25). Compared with other learning styles, accommodating was a style that medical students preferred in this study. Our studies also suggested that students with an accommodating learning style had better perceptions, followed by diverging learning styles. Accommodators studied by feeling and doing and prefer learning from “hands-on” experience (26). Besides, studies indicated that learning style can be related to medical students' academic performance, and teaching methods that matched the learning style that students preferred can promote more effective learning (25, 27). For medical students, therefore, the university can modify teaching methods according to students' learning styles to enhance their perception of MSLE and learning.

Our study determined predictors of medical students' perceptions of MSLE, providing a theoretical basis for elevating students' perceptions of MSLE, thereby improving students' academic performance and physical and mental health. For modifiable factors, such as grade, learning environment of schools, interests in medicine, and Kolb learning experience, investigating effective intervention strategies is necessary. Not only for medical students, but LE was also equally important for other majors students (28). In this study, despite we only explored factors affecting medical students' perceptions of LE, it also provided a reference value for evaluating students' perceptions of LE in other majors.

This study, up to now 20 May 2021, based on a search on PubMed and the Web of Science databases, is the first study to focus on predicting medical students' perceptions of MSLE by constructing nomograms in China. Additionally, this is a large sample study based on multiple centers, so the results are represented to a certain extent. However, this study still has some limitations. Firstly, the establishment of the nomograms is based on the variables determined by the univariate logistic regression analysis, which may lead to overfitting. Secondly, this is a cross-sectional study, so it has certain limitations in verifying causality.

## Conclusion

The construction of the nomograms provides help for teachers to assess medical students' perception levels of MSLE. Gender, university category, grade, mother education level, learning environment of schools, interests in medicine, and Kolb learning experience are associated with medical students' perceptions of MSLE in this study. More large-sample prospective studies should be further carried out to validate the causal relationship between these factors and medical students' perceptions.

## Data Availability Statement

The original contributions presented in the study are included in the article/Supplementary Material, further inquiries can be directed to the corresponding authors.

## Ethics Statement

This study was approved by the Ethics Committee of Tongji Hospital, Tongji University School of Medicine.

## Author Contributions

ZZ, RH, GZ, MG, SX, HY, TM, XW, YW, WC, CZ, ED, ML, XL, QL, SJ, HW, ZH, and JZ: conception, design, collection and assembly of data, data analysis and interpretation, manuscript writing, and final approval of manuscript.

## Funding

This study was supported in part by the National Natural Science Foundation of China (Grant Nos. 81702659, 81772856, and 81801620); Youth Fund of Shanghai Municipal Health Planning Commission (Nos. 2017YQ054 and 2017Y0117); Interdisciplinary Program of Shanghai Jiao Tong University (No. YG2017MS26); Shanghai Talent Development Fund (No. 2018094); Shanghai Municipal Health Commission (No. 201940306); Henan medical science and technology research project (No. 201602031); and Key project of provincial and ministerial co-construction of Henan Medical Science and Technology (No. SBGJ202002031). The funders had no role in study design, data collection and analysis, decision to publish, or preparation of the manuscript.

## Conflict of Interest

The authors declare that the research was conducted in the absence of any commercial or financial relationships that could be construed as a potential conflict of interest.

## Publisher's Note

All claims expressed in this article are solely those of the authors and do not necessarily represent those of their affiliated organizations, or those of the publisher, the editors and the reviewers. Any product that may be evaluated in this article, or claim that may be made by its manufacturer, is not guaranteed or endorsed by the publisher.
