# Supplementary material for: Nomograms for Predicting Medical Students' Perceptions of the Learning Environment: Multicenter Evidence From Medical Schools in China
Source: Front Public Health. 2022 Apr 29;10:825279. doi: 10.3389/fpubh.2022.825279 (PMC9099049; doi:10.3389/fpubh.2022.825279)
Supplement: Supplementary file 1 [file Table_1.docx]

| JHLES item |
| --- |
| **Community of peers** |
| 1. How connected do you feel to other SOM students? |
| 2. How supported do you feel in your personal and professional pursuits by other SOM students? |
| 3. It’s been easy to make friends at the SOM. |
| 4. I feel a sense of community at the SOM. |
| 5. To what extent have you felt a sense of belonging during your time as a student at the SOM? |
| 6. I’ve encountered an abundance of positive, inspiring role models among fellow students at the SOM. |
| **Faculty relationships** |
| 7. I feel that the SOM faculty I encounter are supportive of my professional goals. |
| 8. I feel that SOM faculty members have taken the time to get to know me. |
| 9. I feel that the SOM faculty I encounter genuinely care about my well-being. |
| 10. I’ve encountered an abundance of positive, inspiring faculty role models at the SOM. |
| 11. There are faculty members that I feel comfortable confiding in when important concerns come up. |
| 12. The faculty advisors in the Colleges Advisory Program are readily accessible and interested in students. |
| **Academic climate** |
| 13. Our medical school’s curriculum allows me to use my preferred learning style. |
| 14. I feel that course exams and assessments test my knowledge and abilities fairly. |
| 15. I understand the goals and objectives of the SOM curriculum. |
| 16. To what extent do you trust that the institution has fulfilled your needs as a medical student? |
| 17. The workload during medical school is manageable. |
| **Meaningful engagement** |
| 18. The SOM engages students as meaningful participants. |
| 19. The SOM is flexible and responsive to my needs as a student. |
| 20. I feel that I have a say in decision making about courses and curricular changes. |
| 21. The SOM encourages scholarship and innovation. |
| **Mentoring** |
| 22. I’ve found a mentor in a research field that interests me. |
| 23. I’ve found a mentor in a clinical specialty or discipline that I am passionate about. |
| **Inclusion and safety** |
| 24. I am concerned that students are mistreated at the SOM. |
| 25. I sense there is discrimination based on gender, race, ethnicity, or sexual identity at the SOM. |
| 26. I feel concerned at times for my personal safety at the SOM. |
| **Physical space** |
| 27. The preclinical SOM building has a significant effect on my perception of the learning environment. |
| 28. The work spaces where clinical teaching occurs contributes positively to my sense of the SOM  learning environment. |

Table S1 The 28 items and seven subscales of the JHLES

JHLES, Johns Hopkins Learning Environment Scale; SOM, school of medicine.
